# Supplementary material for: Halide Perovskite glues activate two-dimensional covalent organic framework crystallites for selective NO2 sensing
Source: Nat Commun. 2023 Apr 14;14:2133. doi: 10.1038/s41467-023-37296-0 (PMC10110523; doi:10.1038/s41467-023-37296-0)
Supplement: Supplementary file 1 — Supplementary Information [file 41467_2023_37296_MOESM1_ESM.pdf]

Supplementary Information

Halide Perovskites Glues Activate Two-dimensional Covalent Organic Framework Crystallites for Selective NO_2 Sensing

Wen Ye[1], Liangdan Zhao[2], Hong-Zhen Lin[3], Lifeng Ding[2], Qiang Cao[4], Ze-Kun Chen[4], Jia Wang[4],

Qi-Meng Sun[4], Jing-Hui He[4] and Jian-Mei Lu[1,4]*

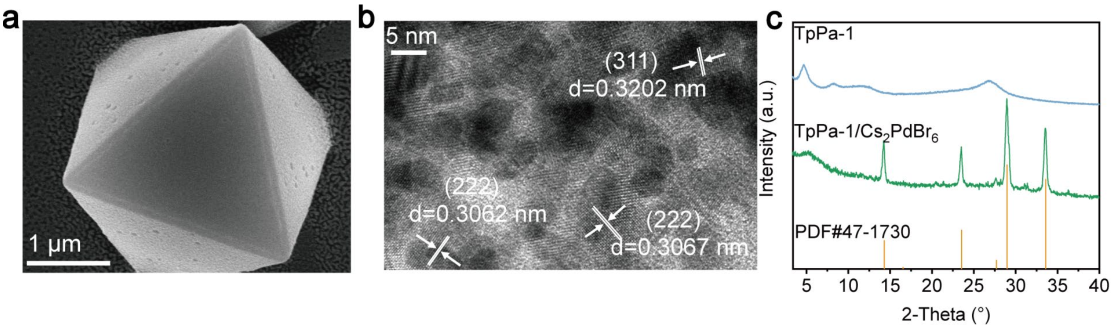

Supplementary Fig. 1: Characterization of the Cs_2PdBr_6 and TpPa-1/ Cs_2PdBr_6. **a** FSEM image and **b** HRTEM image of Cs_2PdBr_6 powders. **c** PXRD pattern of TpPa-1 and TpPa-1/ Cs_2PdBr_6 powders.

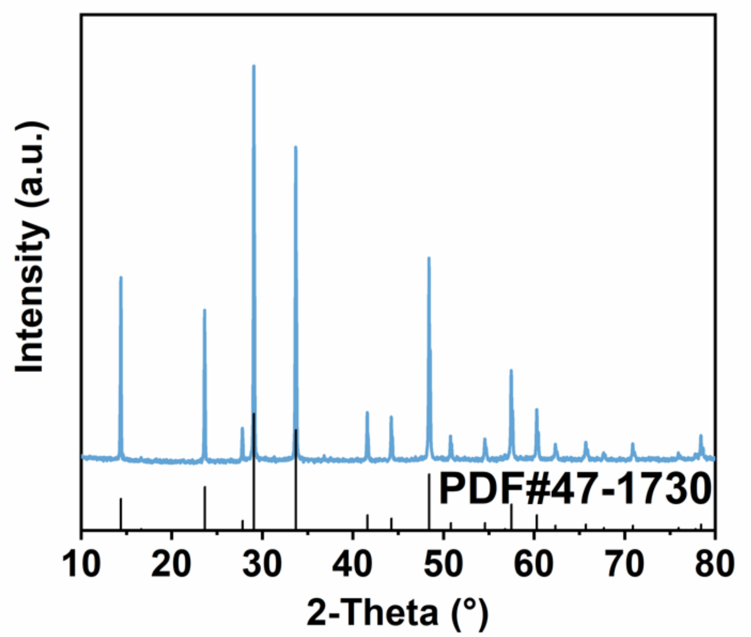

Supplementary Fig. 2: XRD pattern of Cs_2PdBr_6 powders.

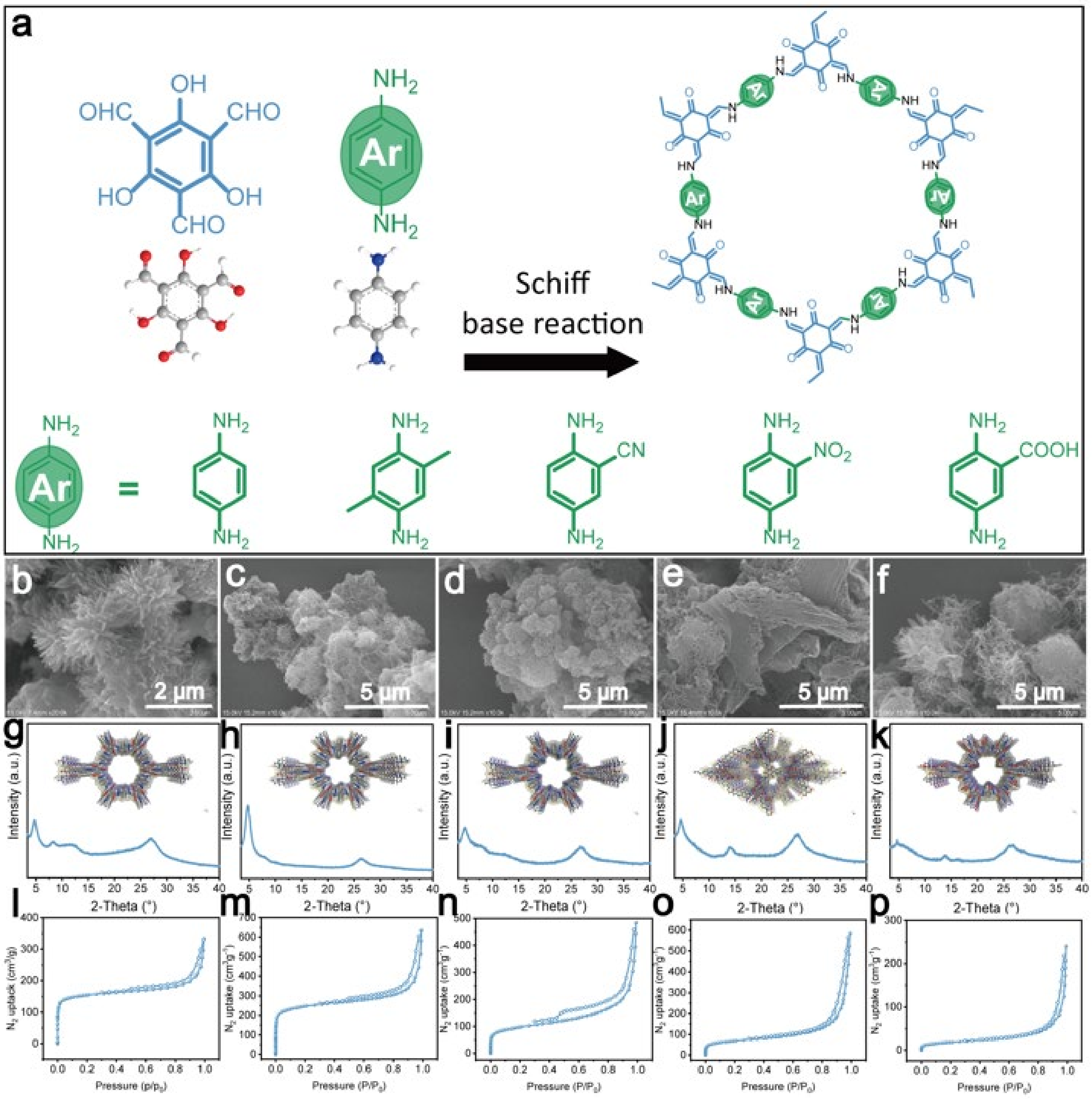

Supplementary Fig. 3: a Synthesis of TpPa series COFs through the Schiff base reactions of 1,3,5-triformylphloroglucinol (Tp) and p-phenylenediamine (Pa-1) and its derivatives (Pa-2, Pa-CN, Pa-NO_2 and Pa-COOH). **b−f** The FESEM images, **g−k** PXRD patterns, **l−p** N_2 sorption isotherms for TpPa-1, TpPa-2, TpPa-CN, TpPa-NO_2 and TpPa-COOH respectively.

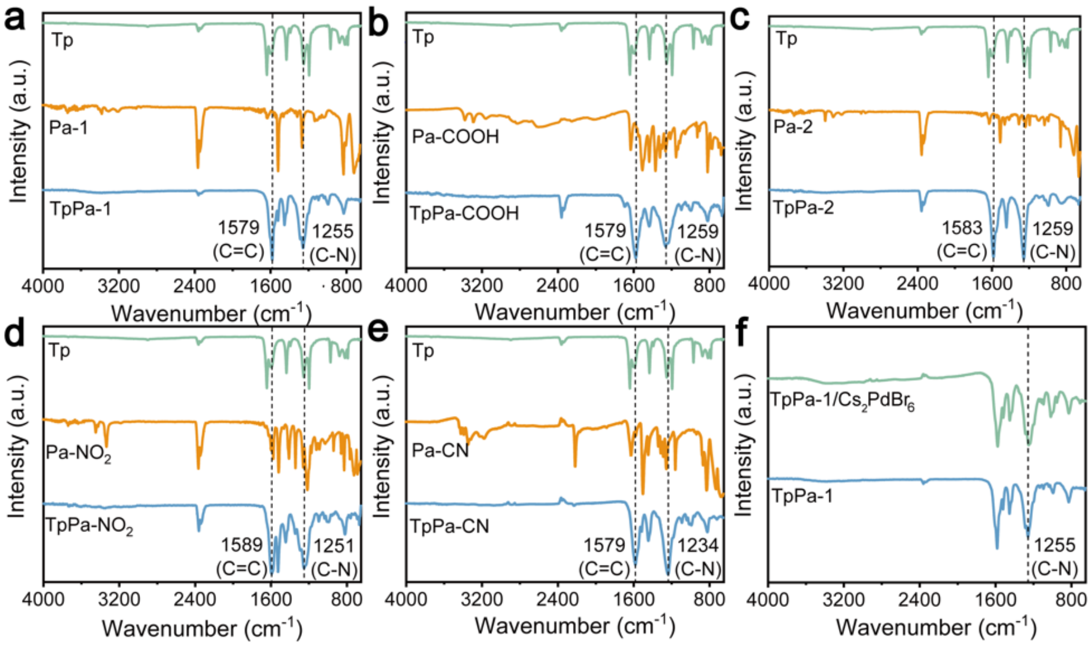

Supplementary Fig. 4: a-e FTIR spectra of different COF and its raw materials. **f** Comparison of FTIR spectra of TpPa-1 and TpPa-1/Cs_2PdBr_6.

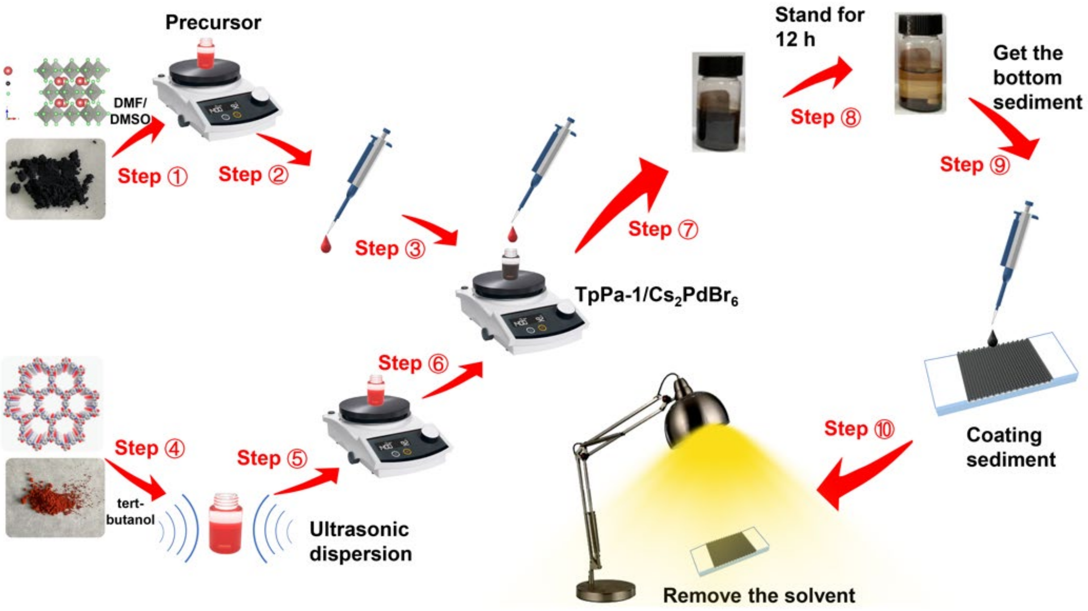

Supplementary Fig. 5: Schematic illustration of the preparation of sensor device.

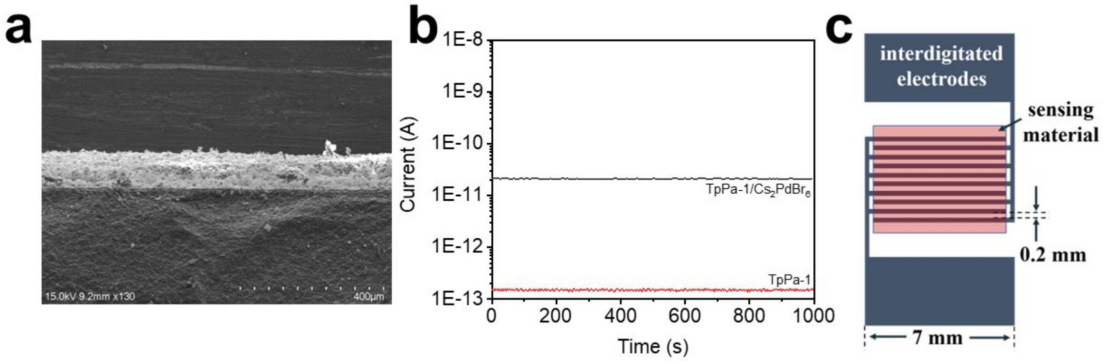

Supplementary Fig. 6: **a** Cross-sectional SEM images of device structure (TpPa-1/Cs_2PdBr_6 film ≈ 100 μm). **b** The change of current with time when the TpPa-1 and TpPa-1/Cs_2PdBr_6 sensor is exposed to N_2 (99.999%). A constant voltage of 5 V was applied across the interdigital electrodes. **c** Specifications of interdigitated electrodes.

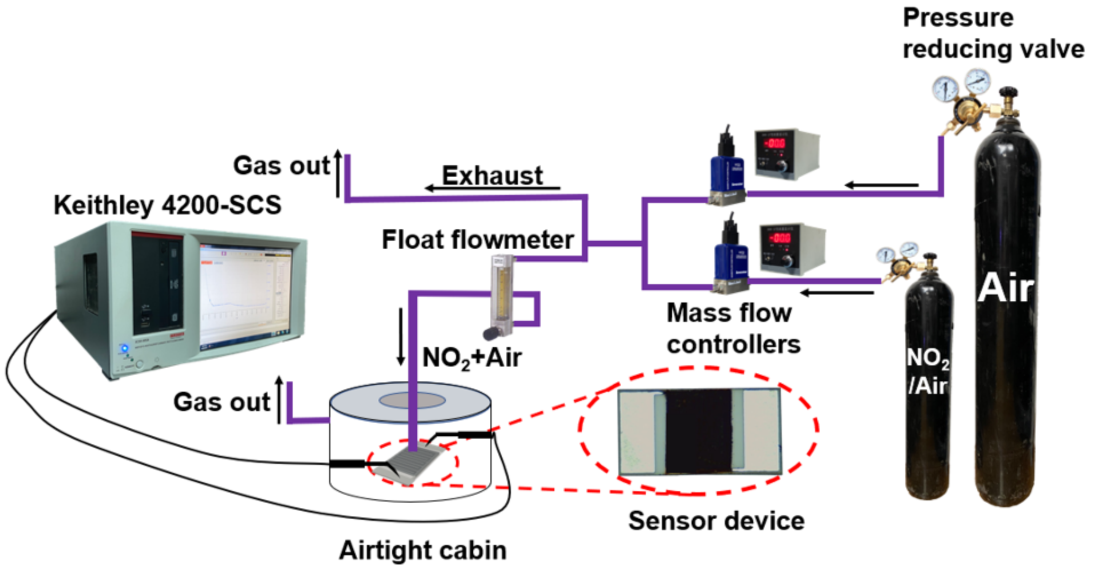

Supplementary Fig. 7: Gas sensor test system.

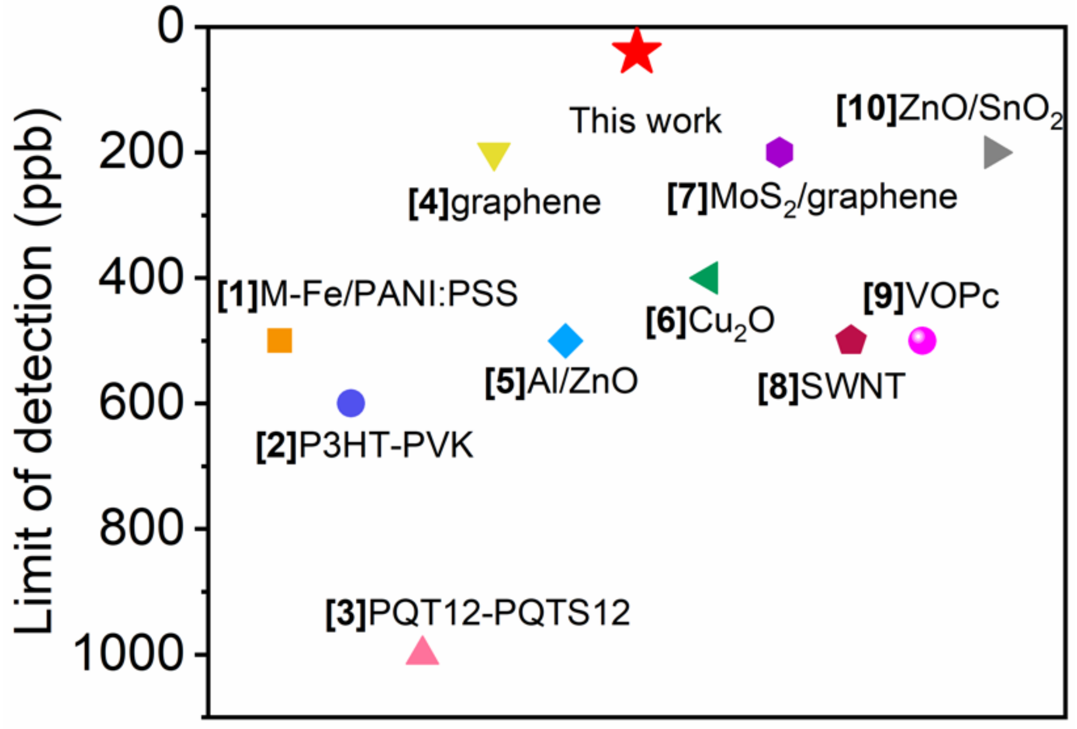

Supplementary Fig. 8: Ranking graph of different NO_2 chemiresistors.[1, 2, 3, 4, 5, 6, 7, 8, 9, 10]

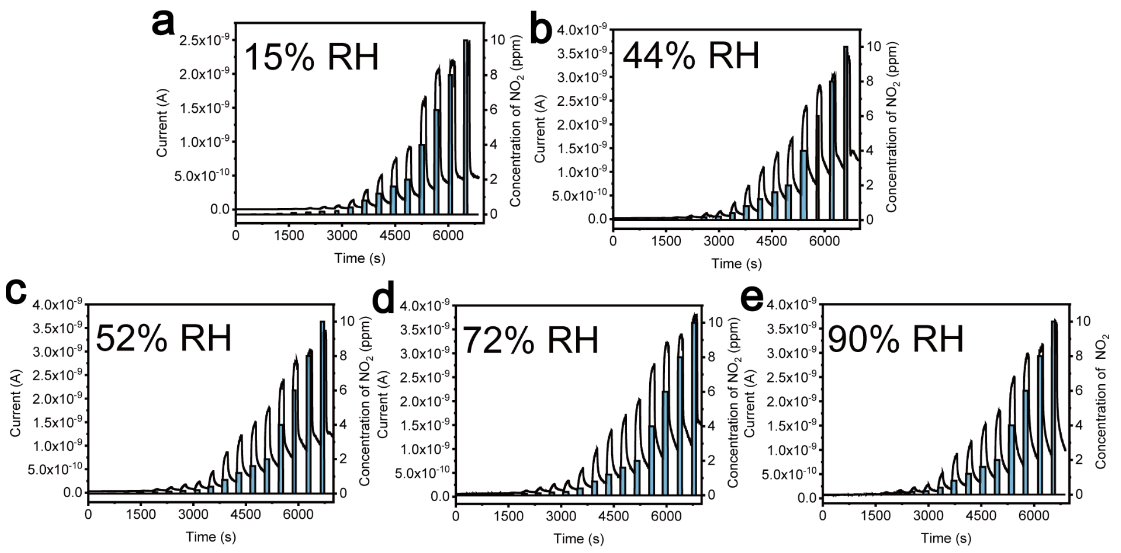

Supplementary Fig. 9: Responses variation versus NO_2 concentrations (40 ppb to 10 ppm) under different humidity conditions.

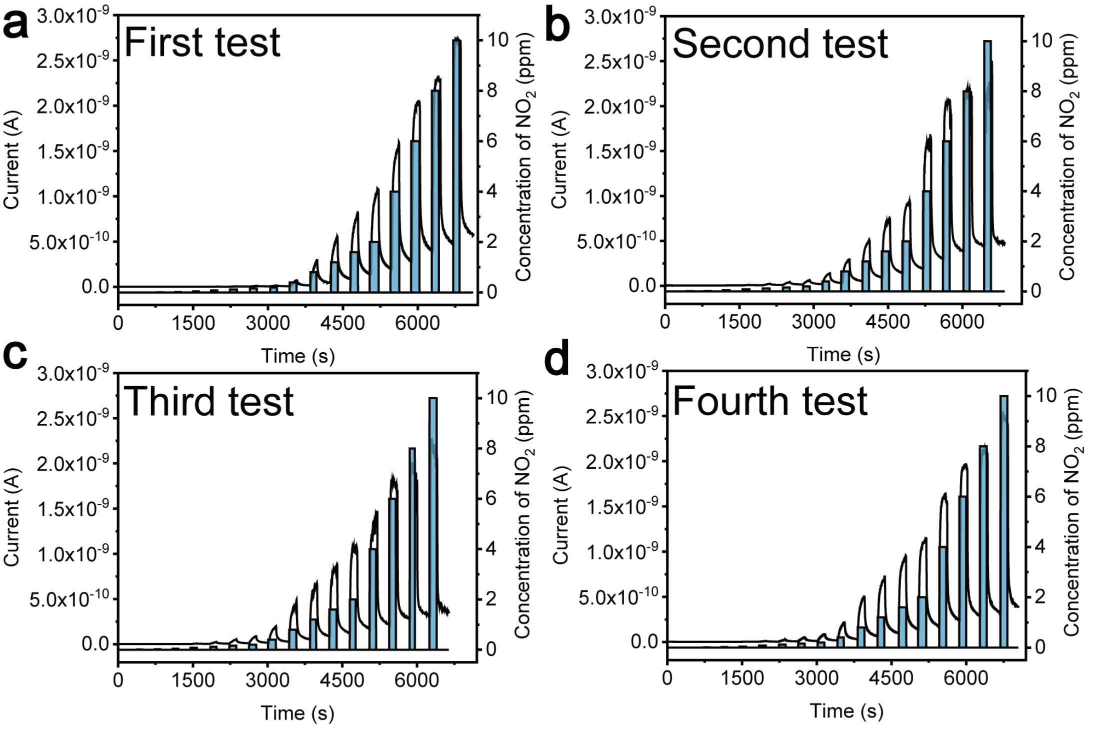

Supplementary Fig. 10: Responses variation versus NO_2 concentrations (40 ppb to 10 ppm) for four times gas sensing test.

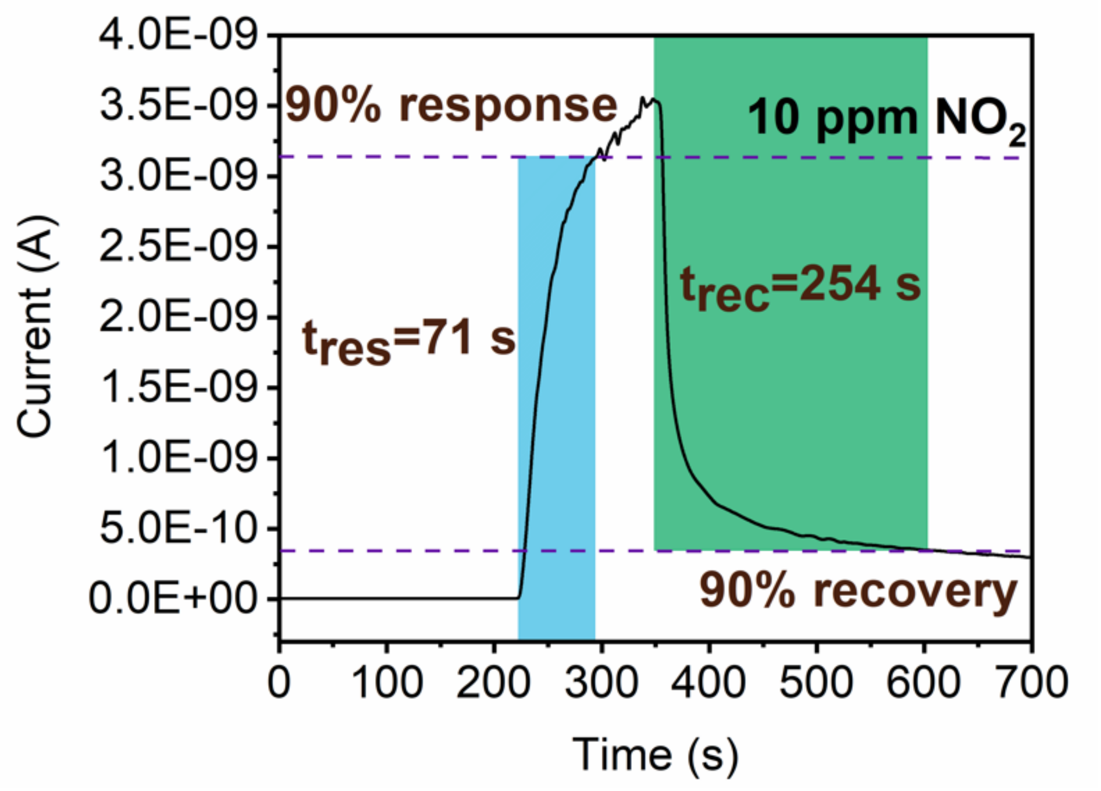

Supplementary Fig. 11: Response/recovery time at 10 ppm NO_2.

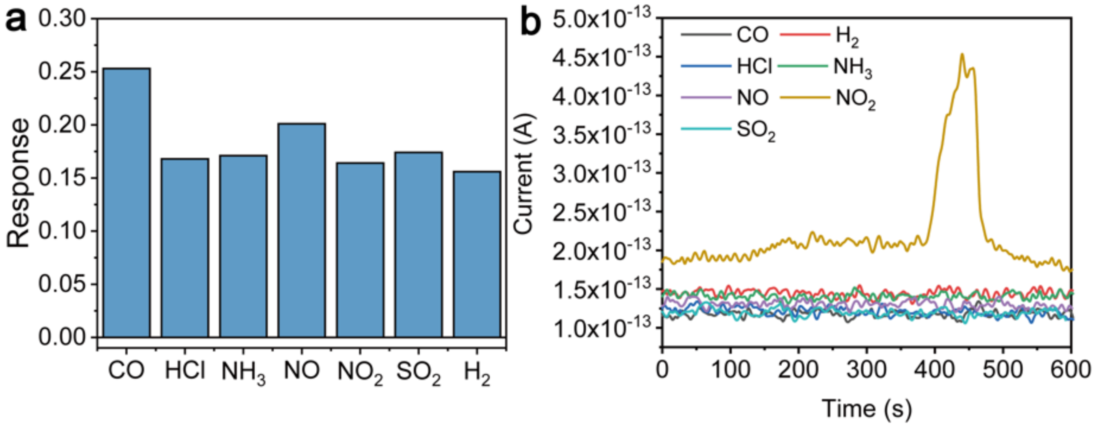

Supplementary Fig. 12: a The response of the Cs_2PdBr_6 sensor to different gases (all at 2 ppm concentration). **b** The change of current with time when the TpPa-1 sensor is exposed to different gases (all at 2 ppm concentration).

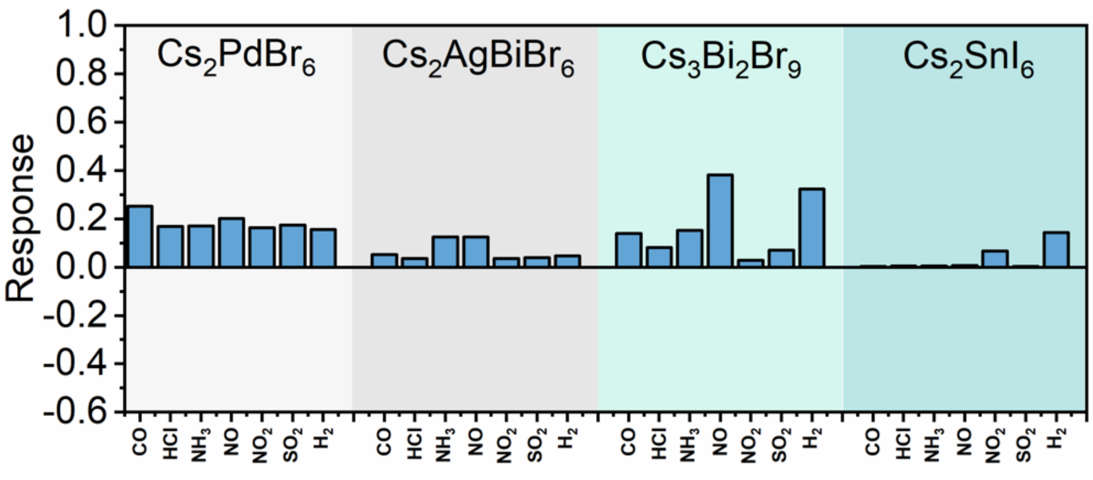

Supplementary Fig. 13: The response of different double perovskite sensors to different gases (all at 2 ppm concentration).

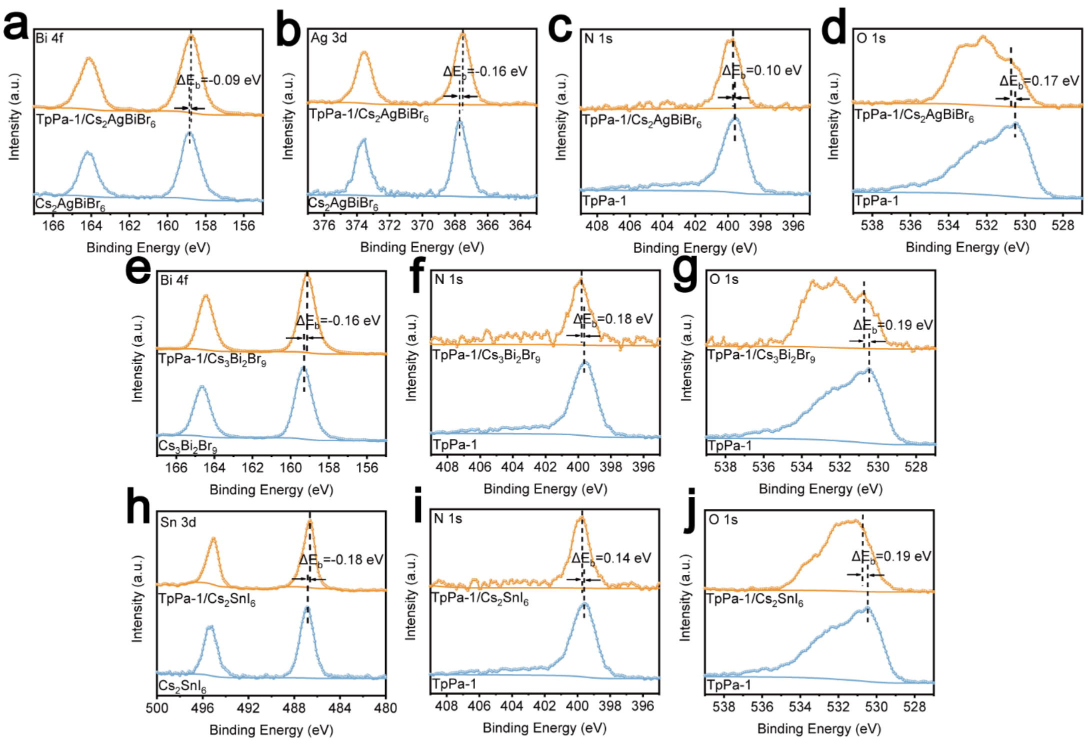

Supplementary Fig. 14: High-resolution XPS spectra of various perovskites mixed with TpPa-1.

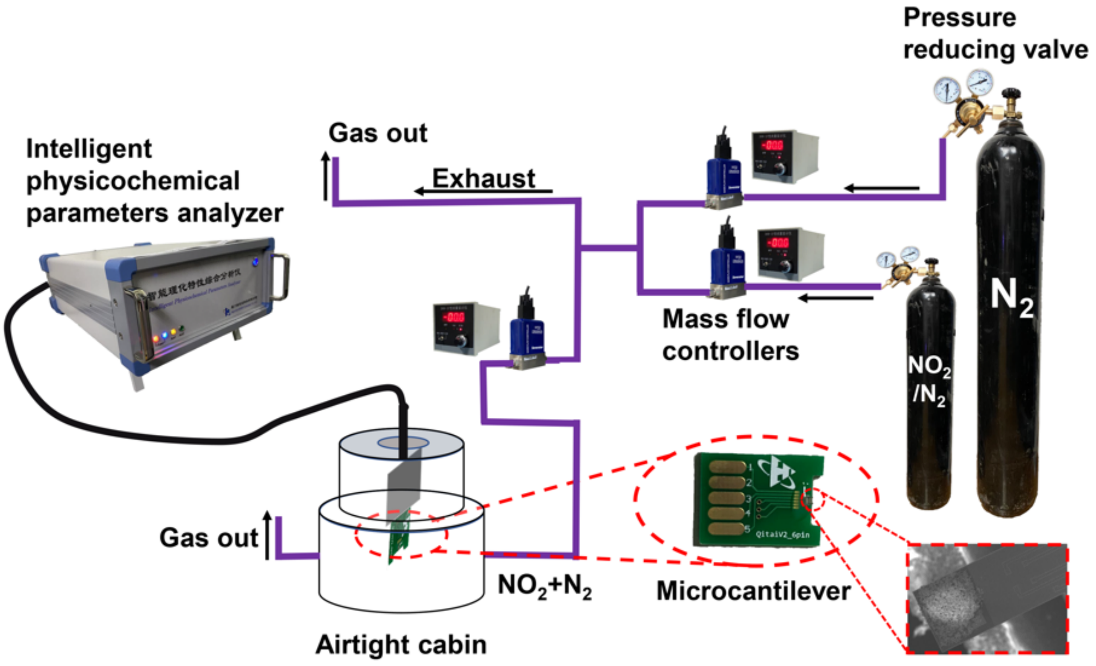

Supplementary Fig. 15: Resonant microcantilever test system.

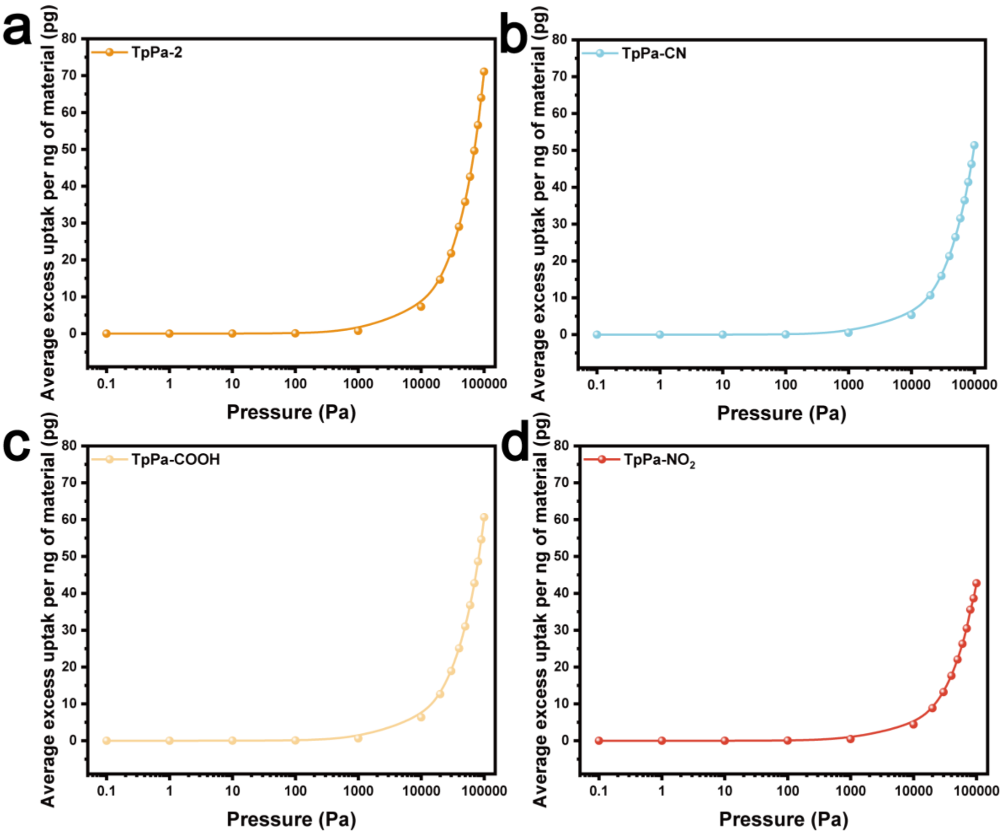

Supplementary Fig. 16: Grand canonical monte carlo (GCMC) simulation of average excess uptake of NO_2/N_2 mixture per ng of material.

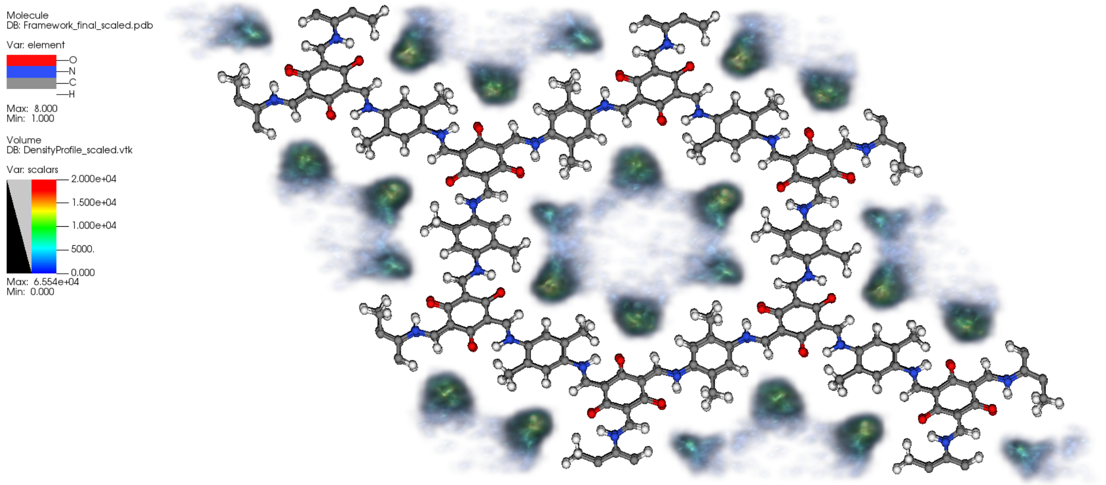

Supplementary Fig. 17: Grand canonical monte carlo (GCMC) simulation of NO_2 adsorption density plot on TpPa-2.

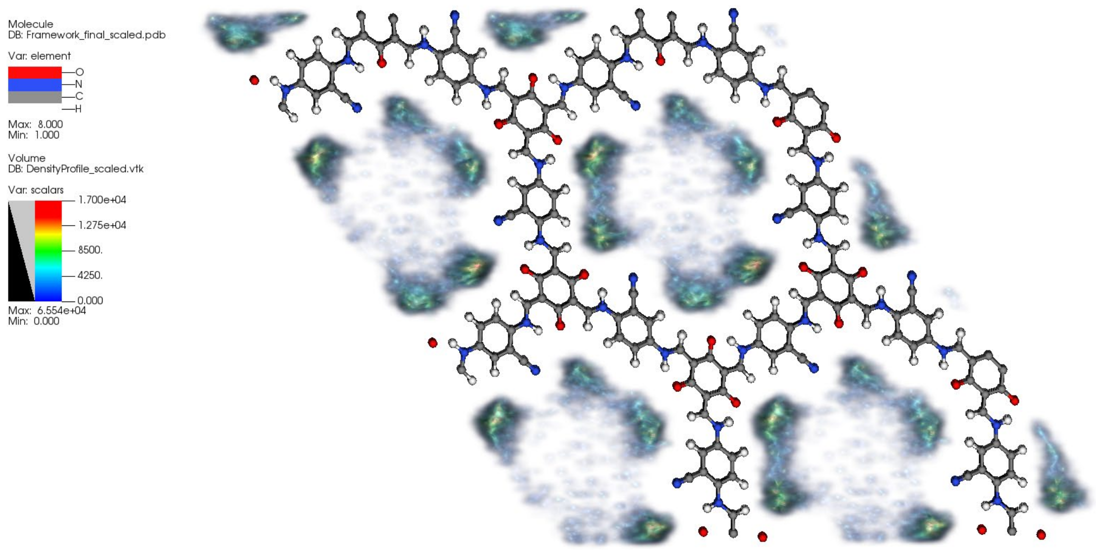

Supplementary Fig. 18: Grand canonical monte carlo (GCMC) simulation of NO_2 adsorption density plot on TpPa-CN.

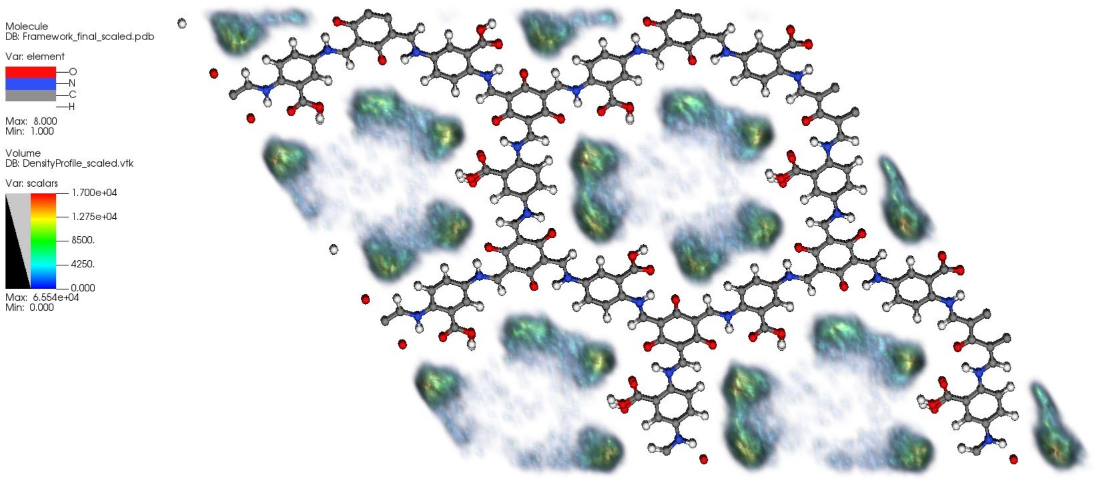

Supplementary Fig. 19: Grand canonical monte carlo (GCMC) simulation of NO_2 adsorption density plot on TpPa-COOH.

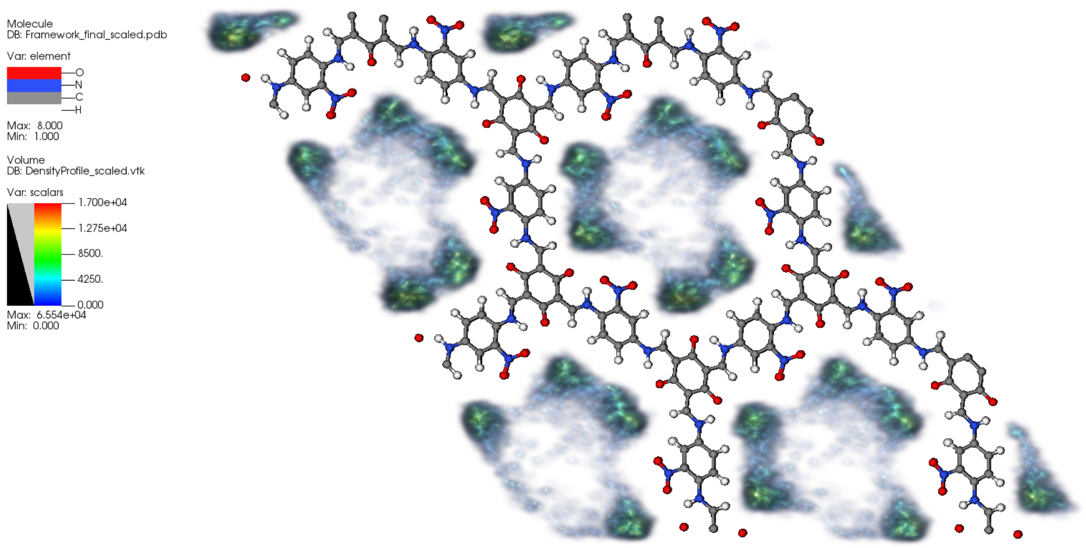

Supplementary Fig. 20: Grand canonical monte carlo (GCMC) simulation of NO_2 adsorption density plot on TpPa-NO_2.

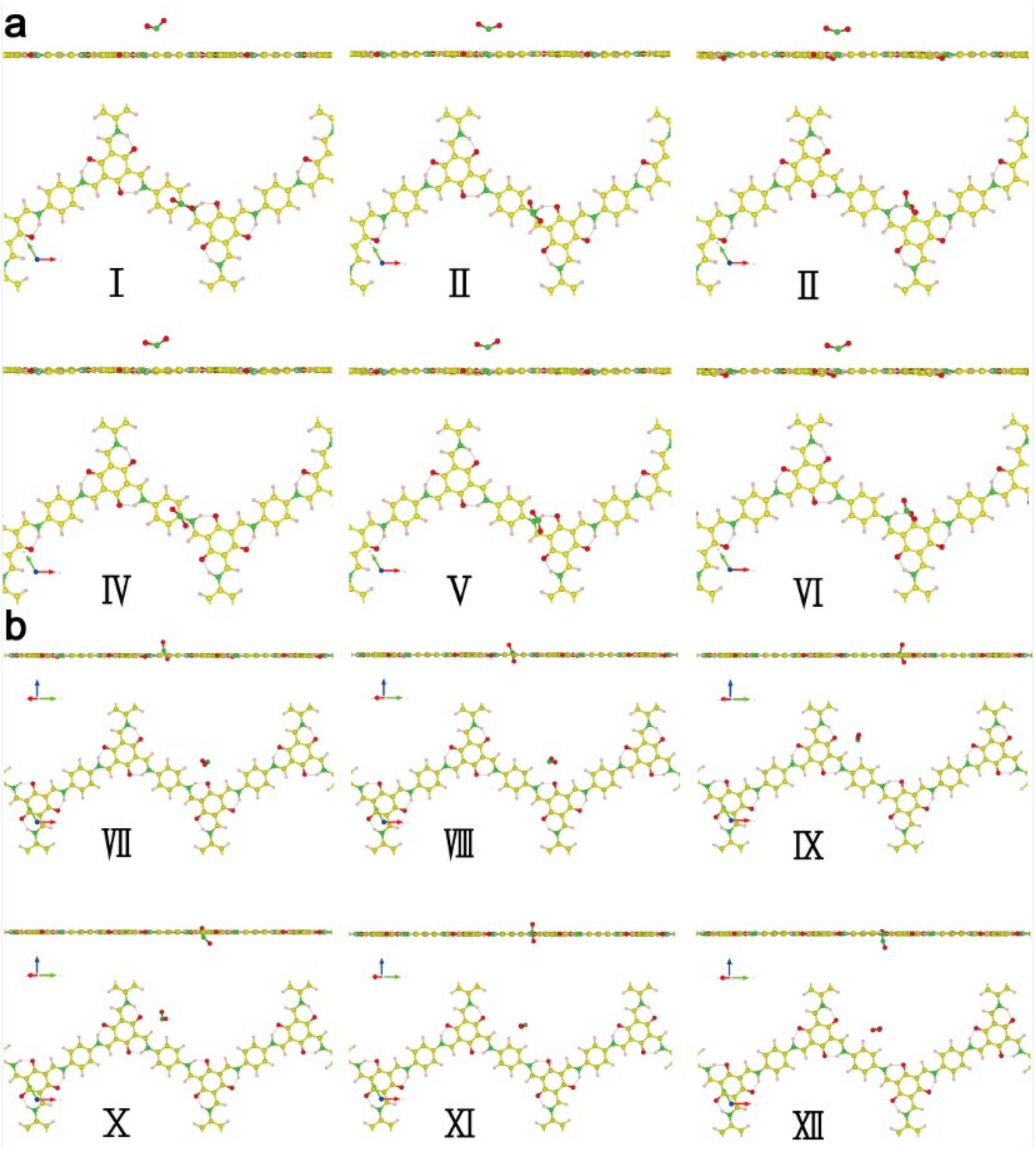

Supplementary Fig. 21: Side and top views of the adsorption configurations for NO_2 (a) on the surface and (b) within the pores of monolayer TpPa-1. The yellow, pink, red and green balls refer to C, H, O and N atoms, respectively.

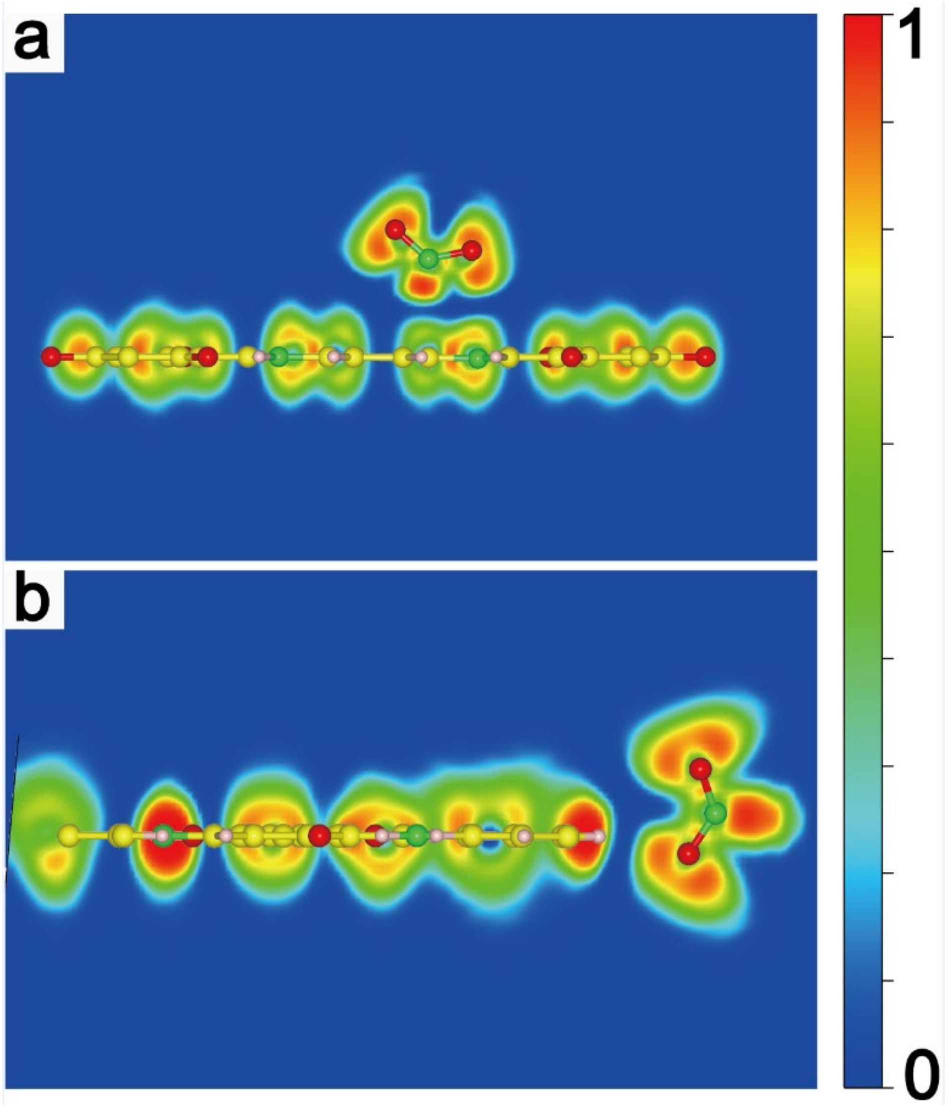

Supplementary Fig. 22: The 2D electron localization function (ELF) of NO_2 adsorbed (a) on the surface and (b) within the pores of monolayer TpPa-1. The yellow, pink, red and green balls refer to C, H, O and N atoms, respectively.

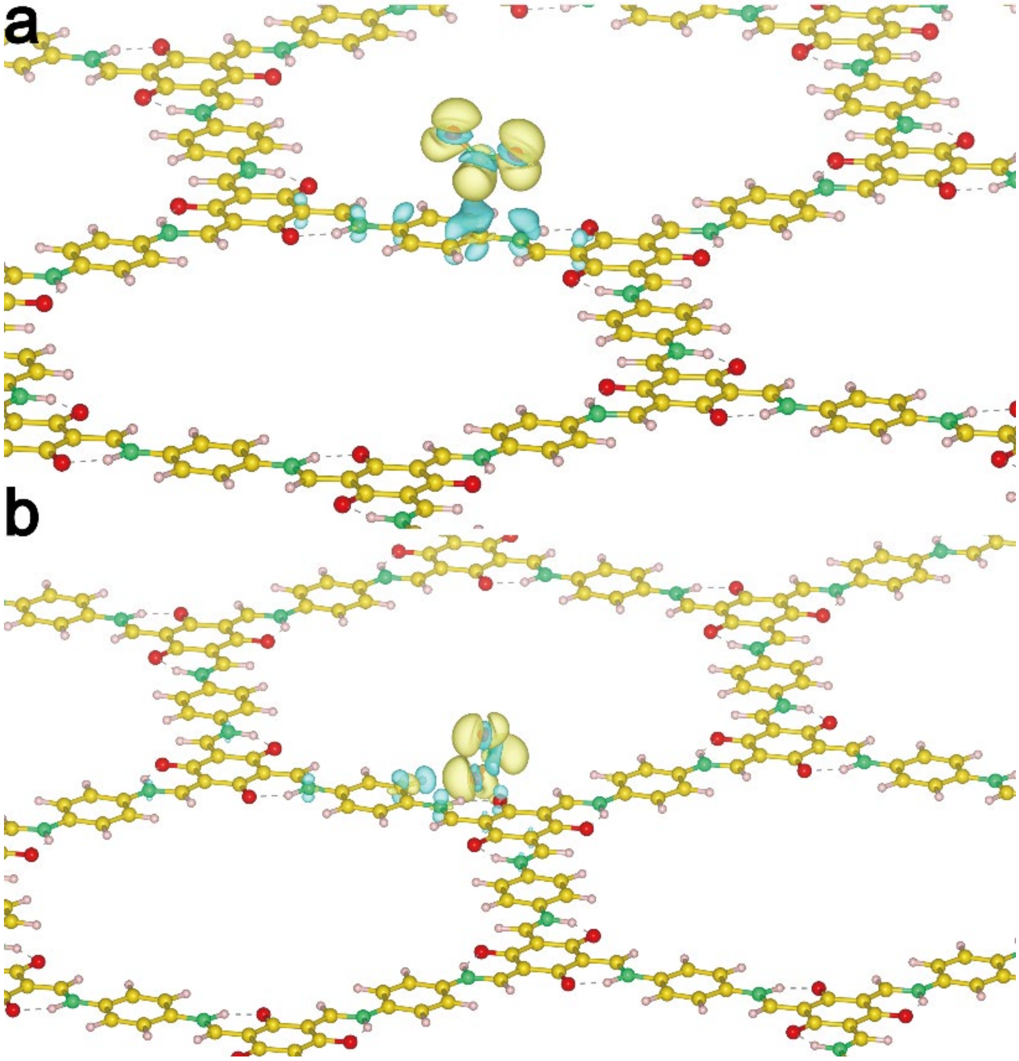

Supplementary Fig. 23: Differential charge density of NO_2. The charge transfer difference between NO_2 and TpPa-1 layers, where the yellow and cyan areas indicate the charge accumulation and depletion with iso-surfaces at 0.007 e/$Å^3$. The yellow, pink, red and green balls refer to C, H, O and N atoms, respectively.

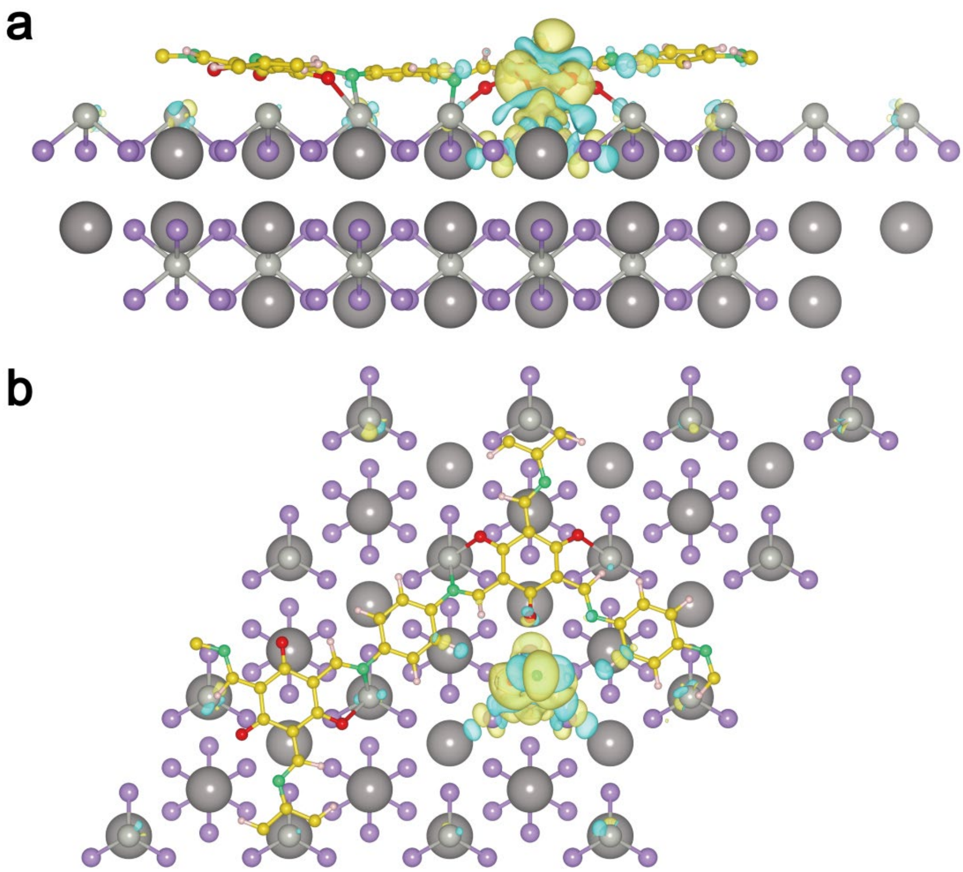

Supplementary Fig. 24: Differential charge density of NO_2. The side and vertical view of the charge transfer difference between NO_2 and TpPa-1/Cs_2PdBr_6 layers, where the yellow and cyan areas indicate the charge accumulation and depletion with iso-surfaces at 0.006 e/Å^3. The yellow, pink, red, green, violet, brown and gray balls refer to C, H, O, N, Br, Pd and Cs atoms, respectively.

Table S1. Total energy and adsorption energy of the corresponding adsorption structures as shown in figure. $E_a = E_{(TpPa\text{-}1\ +\ NO2)} - E_{(TpPa\text{-}1)} - E_{(NO2)}$, $E_{TpPa\text{-}1}$=-507.662 eV, E_{NO2}=-507.18.093 eV

	**I**	**II**	**III**	**IV**	**V**	**VI**
Total energy E (eV)	-526.081	-526.015	-526.047	-526.070	-526.035	-526.052
Adsorption energy E_a (eV)	-0.326	-0.259	-0.292	-0.314	-0.280	-0.297
	**VII**	**VIII**	**IX**	**X**	**XI**	**XII**
Totalenergy E (eV)	-526.042	-526.011	-526.025	-526.037	-526.015	-526.027
Adsorption energy E_a (eV)	-0.287	-0.256	-0.269	-0.281	-0.259	-0.271

References

1. Kim SG, Jun J, Lee JS, Jang J. A highly sensitive wireless nitrogen dioxide gas sensor based on an organic conductive nanocomposite paste. *J Mater Chem A* **7**, 8451-8459 (2019).

2. Han S, Yang Z, Li Z, Zhuang X, Akinwande D, Yu J. Improved room temperature NO2 sensing performance of organic field-effect transistor by directly blending a hole-transporting/electron-blocking polymer into the active layer. *ACS Appl Mater Interfaces* **10**, 38280-38286 (2018).

3. Li H, Dailey J, Kale T, Besar K, Koehler K, Katz HE. Sensitive and selective NO2 sensing based on alkyl-and alkylthio-thiophene polymer conductance and conductance ratio changes from differential chemical doping. *ACS Appl Mater Interfaces* **9**, 20501-20507 (2017).

4. Wu J*, et al.* Facile synthesis of 3D graphene flowers for ultrasensitive and highly reversible gas sensing. *Adv Funct Mater* **26**, 7462-7469 (2016).

5. Sanger A, Kang SB, Jeong MH, Kim CU, Baik JM, Choi KJ. All-transparent NO2 gas sensors based on freestanding Al-doped ZnO nanofibers. *ACS Appl Electron Mater* **1**, 1261-1268 (2019).

6. Deng S*, et al.* Reduced graphene oxide conjugated Cu2O nanowire mesocrystals for high-performance NO2 gas sensor. *J Am Chem Soc* **134**, 4905-4917 (2012).

7. Hong HS, Phuong NH, Huong NT, Nam NH, Hue NT. Highly sensitive and low detection limit of resistive NO2 gas sensor based on a MoS2/graphene two-dimensional heterostructures. *Appl Surf Sci* **492**, 449-454 (2019).

8. Kumar D*, et al.* Effect of single wall carbon nanotube networks on gas sensor response and detection limit. *Sens Actuators, B* **240**, 1134-1140 (2017).

9. Jalil AR*, et al.* Fully integrated organic nanocrystal diode as high performance room temperature NO2 sensor. *Adv Mater* **28**, 2971-2977 (2016).

10. Wang Z, Gao S, Fei T, Liu S, Zhang T. Construction of ZnO/SnO2 heterostructure on reduced graphene oxide for enhanced nitrogen dioxide sensitive performances at room temperature. *ACS Sens* **4**, 2048-2057 (2019).
